# Influence of Running Surface Using Advanced Footwear Technology Spikes on Middle- and Long-Distance Running Performance Measures

**DOI:** 10.3390/sports12120329

**Published:** 2024-12-02

**Authors:** Alejandro Alda-Blanco, Sergio Rodríguez-Barbero, Víctor Rodrigo-Carranza, Fernando Valero, Patricia Chico, Fernando González-Mohíno

**Affiliations:** 1 Sports Training Laboratory, Faculty of Sport Sciences, University of Castilla-La Mancha, 45071 Toledo, Spain; alejandro.alda@uclm.es (A.A.-B.); sergio.rexposito@uclm.es (S.R.-B.); victorrc@tempe.es (V.R.-C.); fernando.valero1@alu.uclm.es (F.V.); patricia.chico1@alu.uclm.es (P.C.); 2 Footwear Innovation Production, TempeInditex, 03203 Alicante, Spain

**Keywords:** super spike, carbon fiber plate, athletics, cross-country running

## Abstract

**Objective:** This study evaluated the effects of advanced footwear technology (AFT) spikes on running performance measures, spatiotemporal variables, and perceptive parameters on different surfaces (track and grass). **Methods:** Twenty-seven male trained runners were recruited for this study. In Experiment 1, participants performed 12 × 200 m at a self-perceived 3000 m running pace with a recovery of 5 min. Performance (time in each repetition), spatiotemporal, and perceptive parameters were measured. In Experiment 2, participants performed 8 × 5 min at 4.44 m/s while energy cost of running (W/kg), spatiotemporal, and perceptive parameters were measured. In both experiments the surface was randomized and mirror order between spike conditions (Polyether Block Amide (PEBA) and PEBA + Plate) was used. **Results:** Experiment 1: Runners were faster on the track (*p* = 0.002) and using PEBA + Plate spike (*p* = 0.049). Experiment 2: Running on grass increased energy cost (*p* = 0.03) and heart rate (*p* < 0.001) regardless of the spike used, while PEBA + Plate spike reduced respiratory exchange ratio (RER) (*p* = 0.041). Step frequency was different across surfaces (*p* < 0.001) and spikes (*p* = 0.002), with increased performance and comfort perceived with PEBA + Plate spikes (*p* < 0.001; *p* = 0.049). **Conclusions:** Running on the track surface with PEBA + Plate spikes enhanced auto-perceived 3000 m running performance, showed lower RER, and improved auto-perceptive comfort and performance. Running on grass surfaces increased energy cost and heart rate without differences between spike conditions.

## 1. Introduction

During running, elastic storage and return of mechanical energy in tendons minimize the metabolic cost of running [1]. However, optimizing external storage and return of energy at the foot–ground interface during ground contact can provide additional metabolic savings [2,3]. Hoogkamer et al. [4] showed that the first advanced footwear technology (AFT) with Polyether Block Amide (PEBA) returned more than twice mechanical energy when compressed with forces similar to vertical ground reaction while running and lowered metabolic cost by 4%, compared to other traditional Ethyl Vinil Acetate (EVA) running shoes. The AFT spikes [spikes that combine lightweight, compliant, resilient foams, i.e., PEBA and a stiff curved (nylon/PEBA/carbon fiber) plate] have recently been shown to improve running economy (RE), expressed as lower energy cost (4–5%) and middle distance performance around 2% running [5,6] similar to their corresponding AFT road shoes [4]. In track races, the performance improvements have been similar, improving more in events where speed is higher [7].

Rodrigo-Carranza et al. [6] showed that the AFT spikes that combined PEBA with a stiff curved carbon fiber plate were the best option to improve the 800 m (∼6.5 m/s) performance. Runners ran faster in PEBA (1.2%) and PEBA + Plate (2.8%) compared to EVA spike. However, PEBA + Plate was better than PEBA condition (1.5%). Nevertheless, RE at 5 m/s was better in PEBA (5.1%) and PEBA + Plate (4.0%) than EVA spike. This suggests that the speed of the track event may influence the design of the best spike for each distance. Moreover, the improvements in both AFT spikes were accompanied by small spatiotemporal modifications, especially in an increase in stride length [5,6].

Historically, spikes have been used in athletics for track events and also for cross-country events that are characterized by courses of 1500 m (short cross or team relay) to 10,000 m in nature with generally soft surfaces such as grass, sand, or woodland courses. These events might also include stretches of gravel paths and hills. In contrast to running on tracks, cross-country surfaces are softer and more deformable. It has been shown that more compliance surfaces with a greater amount of cushioning could improve RE [2,3] with 2.5% slower stride frequency, ~5 cm longer stride length, and 5.9% longer contact time running shod compared to unshod on a rigid treadmill. However, no differences were found running unshod on the treadmill with different amounts of cushioning (10 vs. 20 mm of EVA), which indicates that the cushioning of the surface influences the cushioning of the shoe. Nevertheless, on extremely deformable surfaces, energy cost could increase due to increasing center-of-mass oscillation (vertical oscillation), contact time, and the corresponding decrease in leg stiffness, all of which have been related to a deterioration of RE [8]. This suggests that new AFT with more compliant materials may not improve or even worsen RE/running performance on these types of surfaces.

This raises the question of whether different AFT spikes influence RE and running performance differently depending on the type of surface (track/grass) and the intensity/distance tested. Therefore, the aim of the current study was to examine the influence of different AFT spike components on middle- and long-distance performance measures and spatiotemporal variables on different athletic surfaces. We hypothesized that at middle-distance pace, AFT combined with a modern foam with a curved carbon fiber plate would have the largest improvement in running speed, while PEBA-only would have better RE performance. However, the performance using AFT spikes would be similar between PEBA and PEBA + Plate spikes when running on grass. Small spatiotemporal modifications, such as a reduction in stride frequency, are expected when using PEBA + Plate compared to traditional PEBA spikes with lower differences while running on grass.

## 2. Materials and Methods

### 2.1. Participants

Twenty-seven (Experiment 1: n = 15; Experiment 2: n = 12) trained runners (age: 24.84 ± 5.20 years; body mass: 63.32 ± 6.27 kg; height: 175.73 ± 6.79 cm; performance: World Athletics (WA) score in distance between 5 km and 21 km (score scale used to compare performance across different distances): 779.84 ± 117.05 points) were recruited for this study. The recruitment process was carried out by asking local running clubs. The participants were different in each intervention but with very similar characteristics (Experiment 1: age: 24.11 ± 5.83 years; body mass: 64.00 ± 7.28 kg; height: 175.83 ± 7.24 cm; performance: WA score: 717.00 ± 86.23 points; Experiment 2: age: 25.91 ± 3.92 years; body mass: 62.18 ± 4.30 kg; height: 174.91 ± 6.57 cm; performance: WA score: 861.72 ± 96.69 points).

The inclusion criteria were the following: (1) ranged between 18 and 35 years old; (2) participation in endurance training for at least 5 days per week; (3) <35 min in 10 k event, (4) fitting a men’s 42–44 EU shoe size; and (5) to be a spikes and advanced footwear technology user. The exclusion criteria were the following: (1) not being familiar with AFT spikes, and (2) being injury-free during the previous 6 months. Participants were categorized as nationally trained runners, as they compete at the national level in cross-country, track, and road racing championships [9].

Before this study, all participants were informed about the testing protocols and the possible risks involved; moreover, they were requested to provide written informed consent. This study was performed using the principles of the Declaration of Helsinki (December 2013, Brazil), and the experimental protocols were approved by the Ethics Committee of the local university (CEIC924).

### 2.2. Spike Characteristics

**Figure 1:**
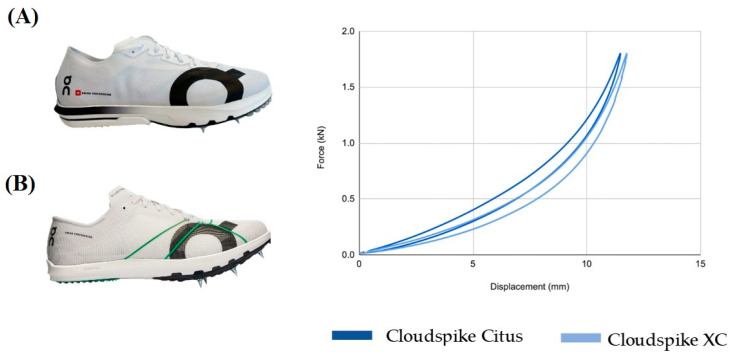
Force–displacement representation for AFT spikes condition used in this study. (**A**) AFT spike with PEBA midsole foam + carbon plate (Cloudspike Citus). (**B**) AFT spike PEBA midsole foam (Clouspike XC).

Two experimental AFT spikes were tested: (1) spikes that combine a curved carbon fiber plate with PEBA midsole foam, and (2) spikes with PEBA midsole foam without elements that increase the longitudinal bending stiffness as a control (Figure 1). All spikes used 6 mm spike pins for traction. The weight of the spikes was 163 g for the PEBA + Plate condition and 142 g for the PEBA condition, both in EU42 (Table 1). Participants could see the spikes they wore but could not manipulate them or learn more about their characteristics.

The force–displacement relationship of the spikes was determined by a compression test on the complete spike (including the shoe upper), where a custom-made structure simulating a size EU42 foot was attached to a material testing machine that measures force and displacement (Zwick/Roell, Ulm-Einsingen, Germany). A force of 1800 N was applied at 2 Hz in the forefoot during 60 cycles, and the average of the last 10 cycles was used for characterizing of spikes. Longitudinal bending stiffness was calculated as the line between 0 and 1500 N.

### 2.3. Experimental Design

Using a counter-balanced randomized experimental design, we evaluated the effects of wearing two AFT spikes, which differ mainly in the midsole design through the insertion of a carbon fiber plate on different surfaces (track and grass). The experimental design consisted of two experiments carried out in different groups (but with homogeneous characteristics) and on different days. All participants were asked to avoid strenuous exercise (no intense exercise in the previous 48 h), caffeine, and alcohol intake 24 h before the visit. The test was carried out on an outdoor athletics track (400 m) with similar environmental conditions in all sessions (529 m altitude, 20–25 °C, and 35–40% relative humidity).

### 2.4. Procedure

#### 2.4.1. Experiment 1: Efforts at Self-Perceived 3000 m Race Pace

**Figure 2:**
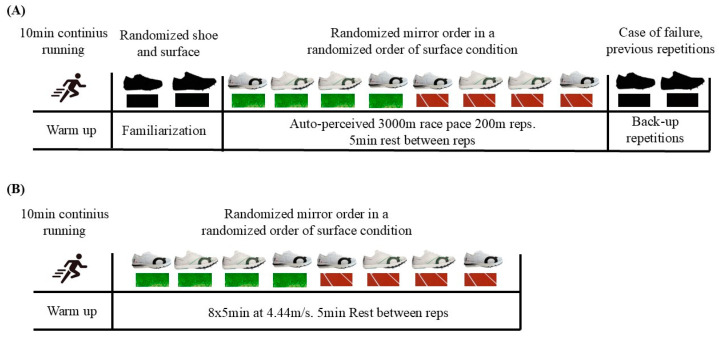
Experimental design. (**A**) Experiment 1: Efforts at self-perceived 3000 m race pace. (**B**) Experiment 2: Running economy protocol at 4.44 m/s.

Warm-up consisted of 10 min of jogging and two runs of 200 m at a self-perceived 3000 m race pace for familiarization. Then, participants performed 12 repetitions of 200 m at auto-perceived intensity [5] (Figure 2), two repetitions with each spike condition and on each surface, in a mirror order in a randomized order of surface condition (i.e., a1–b1–b1–a1–a2–b2–b2–a2). To minimize any confounding effects of participants running the first (excitement) or last (“emptying the tank”) trials at a higher effort, the number of trials that we told the participants to run was greater (12 repeats) than the number of trials they would actually run [5], thus the remaining two repetitions were performed in case of failure of a previous measurement. If the whole procedure was correct, participants performed 10 repetitions. Participants were not informed of the time achieved in each repetition. Rest between repetitions was 5 min. Before each repetition, participants put on the spikes and performed a progressive run of ~100 m toward the starting line to test each spike. Then, they walked the final 100 m to the start line to avoid fatigue.

#### 2.4.2. Experiment 2: Running Economy Protocol at Moderate Intensity

After a standardized warm-up of 10 min of jogging, participants completed 8 repetitions of 5 min at 4.44 m/s (3:45 min/km) (Figure 2). We chose the specific intensity so participants should be able to run below the second ventilatory threshold to ensure steady-state VO_2_ measurement to evaluate RE. They performed two trials in each spike and surface condition, following a mirror order with the surface condition randomized (same as Experiment 1). To ensure that participants maintained a constant pace during each repetition, they were given an acoustic signal each 100 m (split times of 22.5 s for the pace of 3:45 min/km). Rest between repetitions was 5 min, allowing them to change spikes.

### 2.5. Measurements

The main spatiotemporal parameters of the gait cycle (contact time [CT] and step frequency [SF]) were measured for each step during both experiments by using an inertial measurement unit (Stryd Power Meter, Stryd Inc., Boulder, CO, USA) with a sampling frequency of 1000 Hz. The Stryd Power Meter device has shown adequate validity and reliability compared to optical measurement devices and slow-motion recording to measure spatiotemporal parameters [10]. This device does not need any calibration; according to the manufacturer team, it is ready to use out of the box [11].

In Experiment 1, each repetition was recorded using Witty photocells (Microgate, Bolzano, Italy) in an intermediate segment of 25 m. In this way, we ensured that the self-perceived speed measured was accurate. To avoid incorporating the acceleration into the average data, the intermediate time for 25 m was measured on the straight during a segment between 125 and 150 m. The spatiotemporal parameters were measured during that segment.

During the RE efforts (Experiment 2), respiratory variables were measured, specifically those related to gas exchange, including carbon dioxide production (VCO_2_), oxygen consumption (VO_2_), and respiratory exchange ratio (RER), using the Cosmed K5 Wearable Metabolic System (COSMED, Rome, Italy), which was warmed up for a minimum of 30 min calibrated with high-grade calibration gases provided by the manufacturers and by pumping gas with a 3 L calibration syringe through the flow meters, all following the recommendations of the manufacturers. VO_2_ values, collected during the two last minutes of each 5-min trial, were used to calculate RE in W/kg [12]. We expressed RE such as cost (W/kg). The spatiotemporal parameters were measured during the entire effort.

At the end of each repetition with each spike condition in both experiments, participants provided their perception of spike comfort and performance enhancement on a subjective scale [13] from 0 to 100. The questions were: “How comfortable were the spikes?” and “How much do you think the spikes help you during running?”. The corresponding scores for this scale were from 0 = “least comfortable spike I have ever worn” to 100 = “most comfortable spike I have ever worn” and from 0 = “least helpful spike I have ever worn” to 100 = “most helpful spike I have ever worn”, respectively.

### 2.6. Statistical Analysis

Statistical analyses were carried out using IBM SPSS v29.00. (IBM Corp., Armonk, NY, USA) Data were evaluated for normality of distribution and homogeneity of variance using the Shapiro–Wilk test. We assessed all variables normally distributed with a two-way analysis of variance (ANOVA) with repeated measures (spike condition × surface). The sphericity assumption was checked with Mauchly’s test, followed by the Greenhouse–Geisser adjustment where required. The mean of the variables of both experiments was calculated using the two repetitions per spike × surface condition.

Pairwise comparisons with Bonferroni adjustment were used when any significant differences were identified. Results are expressed as mean ± standard deviation. Effect sizes were measured using partial eta squared (η^2^) in the ANOVA analysis, and values of 0.01, 0.06, and above 0.15 were considered as small, medium, and large, respectively [14]. The significance level was set at *p* ≤ 0.05 for all analyses.

## 3. Results

### 3.1. Efforts at Self-Perceived 3000 m Race Pace

**Figure 3:**
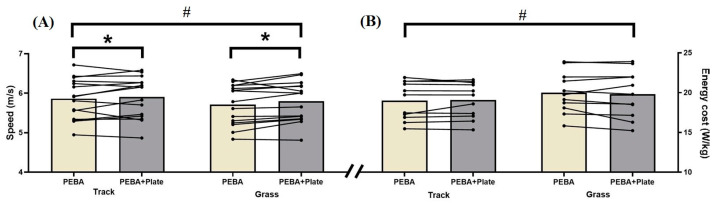
Average speed and energy cost for track and grass repetitions in each spike condition. (**A**) Speed variable in Experiment 1. (**B**) Energy cost variable in Experiment 2. * Significant differences between shoe conditions (*p* < 0.05). # Significant differences between surface conditions (*p* < 0.05).

The results of the efforts at the self-perceived 3000 m race pace are displayed in Table 2 and Figure 3A. There were significant effects for the surface condition (*p* ≤ 0.005; η^2^ = 0.516) and the spike condition (*p* ≤ 0.05; η^2^ = 0.249), without significant differences in the spike × surface interaction (*p* = 0.469; η^2^ = 0.038). The Bonferroni post hoc revealed that speed was faster on the track regardless of spike used (5.882 ± 0.132 vs. 5.742 ± 0.129 m/s, for track and grass, respectively; *p* = 0.002; η^2^ = 0.516) and faster with the PEBA + Plate irrespective of the surface (5.842 ± 0.130 vs. 5.781 ± 0.129 m/s, for PEBA + Plate and PEBA, respectively; *p* = 0.049; η^2^ = 0.249). No significant differences were found between surface or spike conditions for subjective (*p* > 0.05) and spatiotemporal (*p* > 0.05) variables.

### 3.2. Running Economy Protocol at Moderate Intensity

The results of the efforts at moderate intensity are displayed in Table 3 and Figure 3B. There were significant differences in energy cost for the surface factor (*p* ≤ 0.05; η^2^ = 0.391), increasing when running on grass, irrespective of the footwear used (19.911 ± 0.823 vs. 19.03 ± 0.692 W/kg for grass and track, respectively; *p* = 0.03; η^2^ = 0.391). RER presented differences for surface (*p* ≤ 0.001; η^2^ = 0.719) and spike (*p* ≤ 0.05; η^2^ = 0.355) factors; the Bonferroni post hoc revealed that RER was higher on grass regardless of which spike was used (0.92 ± 0.019 vs. 0.88 ± 0.015 RER; for grass and track, respectively; *p* < 0.001; η^2^ = 0.719). Also, PEBA + Plate spike showed less RER in both surfaces (0.903 ± 0.016 vs. 0.91 ± 0.017 RER for PEBA + Plate and PEBA, respectively; *p* = 0.041; η^2^ = 0.355). Heart rate (HR) was significantly lower in track (163.08 ± 2.48 vs. 168.79 ± 2.36 bpm, for track and grass, respectively; *p* = 0.002; η^2^ = 0.616).

Regarding spatiotemporal variables, significant differences were found between surfaces for the SF (*p* < 0.001, η^2^ = 0.688). The Bonferroni post hoc showed a decrease in SF on the track (171.52 ± 1.82 vs. 174 27 ± 2.11 step/min, for track and grass, respectively; *p* < 0.001; η^2^ = 0.688). Furthermore, for the spike factor, significant differences were found for SF (*p* ≤ 0.005, η^2^ = 0.65). SF was lower (172.38 ± 1.92 vs. 173.40 ± 1.98 step/min; *p* = 0.002; η^2^ = 0.650) in the PEBA + Plate spike compared to PEBA spike.

The results of the repeated measures ANOVA showed significant differences in comfort (*p* ≤ 0.05, η^2^ = 0.360) and performance (*p* ≤ 0.001, η^2^ = 0.754) variables for the spike factor. PEBA + Plate was perceived better in both aspects’ (77.37 ± 3.51 vs. 70.25 ± 3.02 Performance; *p* < 0.001; η^2^ = 0.754, 77 ± 4.64 vs. 69.87 ± 3.06 Comfort; *p* = 0.049; η^2^ = 0.360, for PEBA + Plate and PEBA, respectively).

## 4. Discussion

Based on previous literature, AFT spikes could affect RE and performance differently based on the surface type (track or grass) and the intensity or distance being tested. Therefore, the objective of this study was to examine the influence of different AFT spike components (one with PEBA midsole and the other with PEBA midsole with a carbon fiber plate) on middle- and long-distance performance measures and spatiotemporal variables in different athletic surfaces (track and grass). The main findings were that the PEBA + Plate condition was faster in the self-perceived 3000 m race pace (5.842 ± 0.130 vs. 5.781 ± 0.129 m/s; 1.05%; *p* = 0.049; for PEBA + Plate and PEBA, respectively) regardless of the surface (track and grass). However, the energy cost of running was not different between conditions when running at 4.44 m/s, although with a decrease in step frequency in the PEBA + Plate condition (172.38 ± 1.92 vs. 173.40 ± 1.98 step/min; 0.59%; *p* = 0.002)**.**

### 4.1. Differences Between Spike Conditions

Participants ran faster with PEBA + Plate spike (1.05%; *p* = 0.049), regardless of the surface at 3000 m auto-perceived race pace. Previous studies have observed that spikes combining PEBA + Plate were the best choice for improving from 800 m—Mile distance [5,6] and 3000 m performance [6]. Similarly, it appears that combining the PEBA + Plate spikes condition was better than using the PEBA-only condition when running on grass as well. The use of a carbon fiber plate in AFTs has been shown to improve running performance by different mechanisms [15,16]. It seems that, when running at a self-perceived 3000 m race pace, the stiffening caused by the carbon fiber plate minimize energy lost on the metatarsophalangeal joint [17], improving running performance on both surfaces. Also, it optimizes the force–velocity profile in the plantarflexor musculature [18] similar in both surfaces. These mechanics have been shown to result in less muscle activation and lower energy expenditure in cyclic force production movements [19]. However, these mechanisms were not evaluated in our study, so future studies should evaluate why PEBA + Plate spikes work better on any type of surface.

The first AFT studies demonstrated better RE and running performance with AFT compared to traditional racing shoes with EVA midsoles [4,20,21,22,23,24,25]. The improvements in RE were around 4% [4], which initially positioned AFT as the best option for long-distance races (i.e., marathon), where running economy is a key performance factor [26]. When comparing AFT designed for road racing with traditional spikes, it was observed that despite having a higher weight, AFT showed better RE [21]. This prompted brands to develop spikes that incorporate AFT technologies, such as PEBA foam and/or carbon plates. Also, if AFT marathon shoes are compared to AFT spikes, no significant differences were found between them in RE [20], and small changes (0.97%) were found for performance in a time trial of 9 min [27], making AFT better for performance and causing less neuromuscular fatigue [28].

If the comparison is developed between different types of spikes, research has demonstrated that AFT spikes showed better RE when compared with traditional spikes. At 5 m/s, it improved similarly with PEBA (5.1%) and with PEBA + Plate (4.0%) compared to an EVA control spike [6] without significant differences between conditions. Also, at similar speed as the one performed in Experiment 2, significant differences were found between AFT spikes (PEBA-only and thermoplastic polyester elastomer (TPEE)) and control EVA spikes (~2%) [20], but as well as in our research and other previous research, no differences were found between different AFT spikes.

In our study, we did not compare both AFT spikes with a traditional EVA control condition, so we could not evaluate the difference concerning a traditional EVA spike; however, similar to Rodrigo-Carranza et al.’s [6] results, there was no difference between the PEBA and PEBA + Plate conditions. Although in our case the PEBA condition had a lower energy cost (0.42%; *p* = 0.534) running on the track, while the PEBA + Plate condition had a lower energy cost (1.01%; *p* = 0.534) running on grass. When comparing the rest of the metabolic variables, PEBA + Plate condition showed less RER (0.77%; *p* = 0.041) and PEBA condition less HR (0.29%; *p* = 0.175). Which could mean that the distribution of VO_2_ and CO_2_ between conditions was different; however, when taking into account the energy cost, there was no difference between them.

Although differences in performance metrics were observed between various AFT spikes, no differences were detected in energy cost. This leads us to think that at higher speeds PEBA + Plate spikes have better performance, but at moderate efforts there are no differences between AFT spikes with and without a carbon plate. As in previous research, inserting a carbon plate should be considered to develop better performance for middle-distance races, but it is less important for moderate efforts [6]. Therefore, it seems that the effect of speed affects the configuration of the midsole spike components.

When analyzing spike factor for spatiotemporal variables, significant differences were found in Experiment 2, SF decreased (0.59%; *p* = 0.002) with PEBA + Plate spikes. In previous studies about AFT spikes where participants performed 200 m repetitions at an 800 m auto-perceived race pace, AFT spikes showed similar decreased SF than control EVA spikes but without differences between PEBA and PEBA + Plate spikes [6]. In the present study, differences were only found when speed was controlled between spike conditions (controlled pace). In Experiment 1, where participants ran at auto-perceived speeds and significant differences were found between AFT spikes and surface for speed variables, no differences were found for SF variables. The same occurs in the study of Bertschy et al. [5], in which, when the 200 m repetitions with PEBA + Plate spikes were faster, no differences were found in SF. As the speed is significantly higher with PEBA + Plate spikes, the SF should also increase. This does not happen due to the effect this type of footwear has on running mechanics, reducing SF and increasing CT [4]. However, when the speed was the same for both spike conditions (Experiment 2), we observed that SF decreased with PEBA + Plate spikes on the track surface similarly to studies carried out on a treadmill at constant speed where the spatiotemporal changed [29]. This decrease in SF could indicate that stride length has increased proportionally under these conditions. Although no differences were found for CT in any factor in both experiments.

For subjective variables, no differences were found in Experiment 1. However, in Experiment 2, PEBA + Plate was perceived to be more comfortable (9.71%; *p* = 0.049) and better for performance (9.65%; *p* < 0.001). Concerning this, results agree with previous research conducted with advanced footwear technology; spikes with a carbon plate were perceived to improve performance more than those without [30]. In our study, even when changing the surface where the efforts were performed, PEBA + Plate spikes were still perceived to improve performance and comfort more than those without carbon plates.

### 4.2. Differences Between Surface Conditions

In Experiment 1, results showed that athletes ran faster on the track (2.41%; *p* = 0.002), regardless of footwear condition used, and in Experiment 2, for metabolic and cardiovascular variables, results showed that athletes had more energy cost (4.52%; *p* = 0.03), a higher RER (4.44%; *p* = 0.03), and a higher HR (3.44%; *p* = 0.002) at grass at the same speed, regardless of the spike used. The outcomes of this research found that track surfaces reduce energy costs due to their lower compliance compared to grass; this could indicate that track surfaces allow for more efficient energy transfer (i.e., less energy dissipation) [1]. It has already been demonstrated that running on soft surfaces or with cushioned footwear can improve RE [2,3], as long as the cushioning is not excessive. If the deformation of the surface, the footwear, or the combination is too high, it causes an increase in vertical oscillation, a decrease in leg stiffness, and an increase in contact time [8], worsening RE on these types of surfaces [8]. Our results show a significant decrease in SF in Experiment 2 running with PEBA + Plate on the track, while grass repetitions showed higher SF (1.59%; *p* < 0.001).

Therefore, it is important that the cushioning provided by the footwear work with the cushioning provided by the surface. These two aspects must be considered to avoid negatively impacting RE, performance, and spatiotemporal measures.

### 4.3. Practical Application

The results of this research are key for track and cross-country racing; both coaches and athletes need to be informed about the characteristics and intended use of each type of footwear available. For this reason, this article provides essential information to help determine which type of footwear to use based on the surface and the speed of the event. Today, there is a wide variety of models with different characteristics, making it possible to choose and test in sports sciences laboratories the ideal model for each event.

### 4.4. Limitations

This study faced some limitations. The main one could be the self-perceived 3000 m race pace. This approach has been used in a previous study [5] with a shorter distance and faster speed, and the authors found that the approach has the validity, sensitivity, and reliability to quantify the benefits of middle-distance AFT spikes. In addition, we consider our sample to be experienced in this race distance, and it is common to use this approach when prescribing training intensities (e.g., efforts at 1500 m or 10,000 m race pace). However, we understand it is important to point out when interpreting the results. Another limitation was that the experiments were performed only on a male sample, so they could not be extrapolated to women.

## 5. Conclusions

This intervention is the first approach that compares different AFT midsole components and their interaction with the surface (track vs. grass). Results indicate that both surface and spikes models significantly influence performance measures and spatiotemporal variables. PEBA + Plate spikes showed higher speed at a self-perceived intensity of 3000 m compared to PEBA, and this speed was higher on the track regardless of the spikes used. However, no differences between spikes were found for the energy cost of running between spike conditions at moderate intensity. Moreover, the energy cost of running was higher on grass regardless of footwear. Additionally, PEBA + Plate spikes were perceived as more comfortable and effective. In conclusion, both surface and spike types affect performance, with PEBA + Plate spikes showing spatiotemporal changes, mainly with lower SF.

Based on the results of this study, shoe manufacturers should consider designing spikes that combine PEBA midsole with carbon fiber plate to improve performance in middle-distance events on both track and grass/cross-country. However, for longer events where RE is a determining factor, there is no difference between the two spike technologies on any of the surfaces evaluated.

## Figures and Tables

**Table 1:** Characteristics of experimental spikes for EU42.

Characteristics	PEBA + Plate	PEBA
Carbon plate	Full length	No
Midsole foam	PEBA	PEBA
Mass (g)	163	142
Stiffness (N/mm)	155.6	152.1
Energy loss (kN/mm)	1.0	1.1
Energy return (J)	5.7	3.7
Resistance (%)	83.7	82.3
Midsole thickness (mm)	24.5	19.5

**Table 2:** Results of performance, spatiotemporal, and perception variables in the self-perceived 3000 m race pace.

					Repeated-Measures ANOVA					
	Track		Grass		Surface		Spike		Surface × Spike	
	PEBA	PEBA + Plate	PEBA	PEBA + Plate						
Variables	*Mean ± SD*	*Mean ± SD*	*Mean ± SD*	*Mean ± SD*	*p*	η^2^	*p*	η^2^	*p*	η^2^
**Performance**										
Speed (m/s)	5.86 ± 0.513	5.90 ± 0.52	5.70 ± 0.50	5.78 ± 0.50	0.002 *	0.516	0.049 *	0.249	0.469	0.038
**Spatiotemporal**										
Step Frequency (step/min)	194.16 ± 12.42	194.60 ± 13.57	193.00 ± 12.59	193.30 ± 14.32	0.104	0.178	0.551	0.026	0.894	0.001
Contact Time (s)	0.170 ± 0.017	0.165 ± 0.011	0.166 ± 0.080	0.165 ± 0.097	0.536	0.028	0.18	0.125	0.322	0.070
**Perception**										
Performance (0–100)	72.90 ± 17.86	79.03 ± 17.70	71.03 ± 16.33	76.90 ± 18.66	0.248	0.094	0.119	0.165	0.908	0.001
Comfort (0–100)	73.33 ± 21.75	80.07 ± 17.24	74.20 ± 18.18	78.90 ± 20.01	0.95	0.000	0.054	0.241	0.615	0.019

* Statistically significant differences for *p* ≤ 0.05.

**Table 3:** Results of metabolic, cardiovascular, and spatiotemporal variables at moderate intensity.

					ANOVA					
	Track		Grass		Surface		Spike		Surface × Spike	
	PEBA	PEBA + Plate	PEBA	PEBA + Plate						
Variables	*Mean ± SD*	*Mean ± SD*	*Mean ± SD*	*Mean ± SD*	*p*	η^2^	*p*	η^2^	*p*	η^2^
**Metabolic**										
Energy Cost (W/kg)	18.99 ± 2.38	19.07 ± 2.24	20.01 ± 2.57	19.81 ± 2.93	0.030 *	0.391	0.534	0.040	0.435	0.062
RER	0.894 ± 0.050	0.884 ± 0.051	0.926 ± 0.067	0.926 ± 0.061	<0.001 ^#^	0.719	0.041 *	0.355	0.176	0.175
**Cardiovascular**										
Heart rate (bpm)	162.91 ± 7.82	163.26 ± 8.74	168.48 ± 7.86	169.11 ± 7.83	0.002 ^#^	0.616	0.175	0.176	0.522	0.042
**Spatiotemporal**										
Step frequency (step/min)	172.27 ± 5.89	170.77 ± 6.23	174.54 ± 7.35	174.00 ± 6.70	<0.001 ^#^	0.688	0.002 *	0.650	0.077	0.280
Contact time (s)	0.189 ± 0.015	0.188 ± 0.013	0.188 ± 0.011	0.191 ± 0.014	0.617	0.026	0.659	0.020	0.371	0.081
**Perception**										
Performance (0–100)	70.00 ± 11.61	78.25 ± 9.65	70.50 ± 10.92	76.50 ± 14.15	0.735	0.013	<0.001 ^#^	0.754	0.710	0.016
Comfort (0–100)	70.25 ± 8.20	77.25 ± 14.88	69.50 ± 14.03	76.75 ± 16.83	0.858	0.004	0.049 *	0.360	0.952	0.000

* Statistically significant differences for *p* ≤ 0.05; # statistically significant differences for *p* ≤ 0.001.

## Data Availability

The datasets used and/or analyzed during the current study are available from the corresponding author upon reasonable request.
